# Antibacterial Activity of Ebselen

**DOI:** 10.3390/ijms24021610

**Published:** 2023-01-13

**Authors:** Marta Maślanka, Artur Mucha

**Affiliations:** Department of Bioorganic Chemistry, Wrocław University of Science and Technology, Wybrzeże Wyspiańskiego 27, 50-370 Wrocław, Poland

**Keywords:** organoselenium compounds, repurposing, antimicrobials, covalent drugs, bacterial proteins, cysteine modification, mechanisms of inhibition

## Abstract

Ebselen is a low-molecular-weight organoselenium compound that has been broadly studied for its antioxidant, anti-inflammatory, and cytoprotective properties. These advantageous properties were initially associated with mimicking the activity of selenoprotein glutathione peroxidase, but the biomedical impact of this compound appear to be far more complex. Ebselen serves as a substrate or inhibitor with multiple protein/enzyme targets, whereas inhibition typically originates from the covalent modification of cysteine residues by opening the benzisoselenazolone ring and S–Se bond formation. The inhibition of enzymes of various classes and origins has been associated with substantial antimicrobial potential among other activities. In this contribution, we summarize the current state of the art regarding the antibacterial activity of ebselen. This activity, alone and in combination with commercial pharmaceuticals, against pathogens, including those resistant to drugs, is presented, together with the molecular mechanism behind the reactivity. The specific inactivation of thioredoxin reductase, bacterial toxins, and other resistance factors is considered to have certain therapeutic implications. Synergistic action and sensitization to common antibiotics assisted with the use of ebselen appear to be promising directions in the treatment of persistent infections.

## 1. Introduction

**Figure 1:**
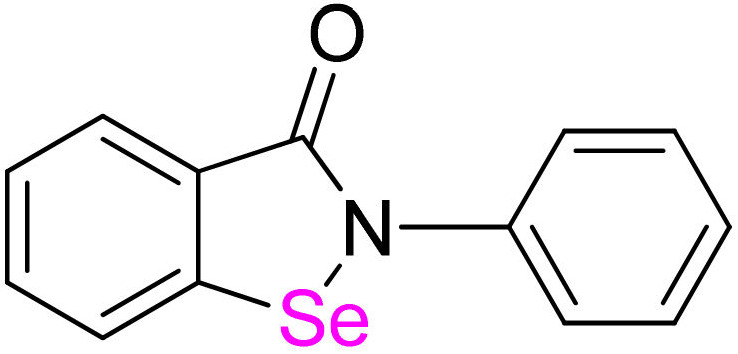
The chemical structure of ebselen.

Bacterial resistance to antimicrobials has become a significant global health threat. According to the most systematic estimate, 1.27 million deaths in 2019 could be directly attributed to antimicrobial resistance, while 4.95 million fatal cases were associated with antimicrobial resistance [1]. Comprehensive accomplishments, such as investments in new therapies, worldwide infection control, and balanced use of existing antibiotics, are in rapid demand, but they cannot change the fact that the process of drug discovery and development of innovative antimicrobials suffers from decades of missing breakthrough. The urgency for efficient treatments can be circumvented in the short term by alternative initiatives, such as drug repurposing [2,3]. The concept of identifying new targets and uses for known drugs/bioactive compounds opens opportunities for serendipitous and systematic approaches to discovery processes. Ebselen (2-phenyl-1,2-benzisoselenazol-3(*2H*)-one, C_13_H_9_NOSe, MW 274.18 g/mol, m.p. 180–181 °C, white or yellowish crystalline solid, soluble in DMSO, poorly soluble in water, Figure 1), an antioxidant, anti-inflammatory, antiatherosclerotic, and cytoprotective organoselenium compound [4,5,6,7] has recently become one of the leading compounds with repurposing potential. It shows antibiotic (but also antiviral, antifungal, and antiprotozoal) properties alone and in combination with existing drugs [4,5,6,7,8,9]. These properties are attributed to the multifaceted reactivity of ebselen with protein thiols. In this paper, we present recent achievements in the application of ebselen as an antimicrobial, with particular attention to its action at the molecular level.

## 2. Antibacterial Activity of Ebselen against Drug-Resistant Bacteria

The antibacterial properties of ebselen were originally verified by Nozawa et al. with a series of Gram-negative and Gram-positive clinical isolates resistant to methicillin, a narrow-spectrum β-lactam antibiotic of the penicillin class [10]. In general, ebselen was found to be active or moderately active in Gram-positive bacteria and poorly active in Gram-negative strains. Consequently, the growth of strains of *Staphylococcus aureus* and *Staphylococcus epidermidis* were inhibited at a concentration of 0.20 µg/mL and the streptococcal species at 1.56–6.25 µg/mL, while members of the Enterobacteriaceae family were more resistant (12.5–50 µg/mL). The presence of the selenium atom was shown to be essential for the antibacterial activity because the corresponding sulfur analog lost the potency of the organoselenium counterpart. The authors correlated the ebselen/ebsulfur potency ratio with their antioxidant properties, which were principally relevant to the chemical reactivity with sulfur nucleophiles.

Recently, the repurposing strategy confirmed ebselen as a potent antimicrobial agent by selecting it from a 727-member library of nonantibiotic drugs (including those approved by the FDA) [11]. The library was tested against six pathogens (*Enterococcus faecium*, *S. aureus*, *Klebsiella pneumoniae*, *Acinetobacter baumannii*, *Pseudomonas aeruginosa*, and *Enterobacter cloacae*—ESKAPE), and ebselen demonstrated clinically applicable bactericidal activity (minimum inhibitory concentration, MIC = 0.25 μg/mL) with *S. aureus* isolates resistant to methicillin and vancomycin, a glycopeptide antibiotic.

Further detailed studies demonstrated potent bactericidal activity against a range of clinical isolates of methicillin-, vancomycin- and other multidrug-resistant Gram-positive pathogens [12,13]. Minimum inhibitory concentrations ranged from 0.0625 to 1 μg/mL (with the majority between 0.125–0.5 μg/mL) for the strains of Staphylococcus, Streptococcus, and Enterococcus regardless of their resistance phenotype. Activity outscored the potency of vancomycin and linezolid (an oxazolidinone, which suppresses the production of bacterial protein and is the drug of choice for Enterococcus and Staphylococcus infections) [12]. Ebselen was also shown to have extracellular and intracellular antistaphylococcal activity as it reduced methicillin-resistant *S. aureus* in murine macrophage-like J774A.1 cells considerably more than vancomycin and linezolid. The discovery of dual extra- and intracellular activity was of a great significance as extracellular pathogens, such as *S*. *aureus*, could invade and survive in the mammalian host cells. The treatment of such persistent and chronic infections is problematic and limited by capabilities of passing antibiotics through cellular membranes. Ebselen was found to not be cytotoxic (IC_50_ = 96 μg/mL) against the J774A.1 strain. The in vitro activity corresponded to excellent potency in vivo in the whole animal *Caenorhabditis elegans* infected with methicillin-resistant *S. aureus*. The drug reduced the mean bacterial count by 85% at a concentration of 8 μg/mL. It acted synergistically with conventional antimicrobials, such as linezolid, clindamycin, vancomycin, chloramphenicol, erythromycin, rifampicin, and gentamicin [12].

The investigation of the mechanism of action of ebselen by the incorporation of radiolabeled precursors demonstrated that the compound inhibited the synthesis of bacterial proteins at MIC and nucleic acids and lipids at higher concentrations (8×MIC) [13]. Following the inhibition of protein biosynthesis in a strain resistant to methicillin, ebselen suppressed the production of two important cytotoxins, α-hemolysin, and Panton-Valentine leucocidin after 1 h of incubation. Additionally, at 16×MIC concentrations (2 μg/mL and 8 μg/mL, respectively), the mass of biofilms formed by *S. aureus* and *S. epidermidis* was reduced by 50–60%, which was much more effective than the action of reference antibiotics, such as linezolid, mupirocin, vancomycin, and rifampicin. In a mouse model of methicillin-resistant *S. aureus* skin infection, ebselen showed therapeutic potential. At 1–2% concentrations, it substantially reduced the bacterial load and the levels of proinflammatory cytokines. It acted synergistically with topical antimicrobials (mupirocin, fusidic acid, retapamulin, and daptomycin) against clinical isolates. Toxicity measured with human keratinocyte cells was at a tolerable level with IC_50_ = 59 μg/mL.

In continuation, the in vitro activity of ebselen against vancomycin-resistant enterococcal isolates was compared to linezolid and ramoplanin, a glycolipodepsipeptide that inhibits cell wall biosynthesis. Ebselen and linezolid inhibited the growth of strains at a concentration of 0.25–4 μg/mL (typically, 0.5–2 μg/mL), while ramoplanin was twice as potent [14]. A standard time–kill assay identified the bacteriostatic properties of ebselen and linezolid against vancomycin-resistant *E. faecium* at two concentrations (3×MIC and 6×MIC) and the rapid bactericidal action of ramoplanin. Fourteen consecutive passages did not induce the development of the resistance of the *E. faecium* strain to the organoselenium drug (no change in MIC). The compound studied inhibited biofilm formation in a concentration-dependent manner, but ebselen appeared to be superior in eradicating mature biofilms (95% at 16×MIC). Finally, the treatment of mice colonized with vancomycin-resistant *E. faecium* with ebselen and ramoplanin significantly reduced the burden on the contents of the gastrointestinal tract [14].

The antimicrobial potential of ebselen was compared with linezolid and mupirocin against two methicillin-resistant strains of *S. aureus* in the topical treatment of infected pressure ulcers using obese and diabetic mice [15]. In obese mice, ebselen reduced the burden by 89.2% and linezolid reduced it by 84.5% while mupirocin was found to be superior (98.7%). In diabetic mice, ebselen was much less effective than mupirocin (45.8% versus 99.3% reduction). Therefore, higher doses and longer treatment would be needed to achieve the corresponding effects. Interestingly, *S. aureus* did not develop resistance to ebselen after 14 consecutive exposures; in contrast, resistance to mupirocin emerged rapidly (MIC increased by at least two orders of magnitude after 5–6 passages) [15].

The inhibitory and bactericidal properties of four nonantibiotics, including ebselen, against five clinical isolates of *S. aureus* obtained from patients with skin and soft tissue infections were also presented by Boyd et al. [16]. The authors demonstrated varying in vitro activity, with ebselen being the most potent compound compared to amlodipine, a calcium channel blocker (MIC = 64 μg/mL); azelastine, an antihistamine (MIC = 200 μg/mL); and sertraline, a selective serotonin reuptake inhibitor (MIC = 20 μg/mL). The MICs for ebselen varied from 0.25 to 1.0 μg/mL, outperformed the other three nonantibiotics, and were comparable with the most active among nine reference antibacterial drugs (oxacillin, clindamycin, erythromycin, gentamicin, trimethoprim/sulfamethoxazole, doxycycline, tetracycline, vancomycin, and ciprofloxacin). The minimum bactericidal concentrations of ebselen were within one dilution of their MICs for all isolates tested [16].

It is well established that the resistance of bacterial infections to common antimicrobials is often caused by the formation of biofilms and matrix-enclosed bacterial communities [17]. The repurposing strategy of 1280 commercially available drugs (off-patent drugs at the Prestwick Chemical Library), some with previously unknown antimicrobial activity, was used to identify new compounds active against *S. aureus* [18]. Again, ebselen was classified as an effective agent against planktonic strains of *S. aureus* (IC_50_ = 1.3 µM) together with 17 other selected non-antimicrobials. However, in these studies, ebselen was not selected for tests against the preformed biofilms of *S. aureus*. It seemed unfortunate as it efficiently eradicated the biofilms of *S. aureus*, *S. epidermidis* [13], and *E. faecium* [14], as mentioned above.

The bactericidal properties of ebselen, manifested by an elevated level of reactive oxygen species and leading to cell death, are currently used to treat mature biofilms of *Neisseria mucosa* [19]. The action of ebselen involved the degradation of the extracellular polymeric layer and attenuation of quorum sensing by the inhibition of particular hydrolases (urease and proteases).

Zou et al. constructed an advanced antibiofilm nanodelivery system against *Helicobacter pylori* [20]. Nanoparticles consisted of self-assembled linoleic acid and an *N*-biguanidylethyl derivative of linoleic amide encapsulating ebselen and coated with fucoidan polysaccharide. The negatively charged system was designed to easily penetrate the gastric mucus layer and infection site. After the elimination of extracellular polymeric substances from the structure of the *H. pylori* biofilm, the nanoparticles were able to penetrate the cells using surface fucoidan-binding proteins. Finally, the elevation of oxidative stress, inhibition of urease with ebselen (*vide infra*), and stimulation of 5′ AMP-activated protein kinase activity in host cells led to pathogen cell death.

## 3. Inhibition of Bacterial Thioredoxin Reductase

**Scheme 1:**
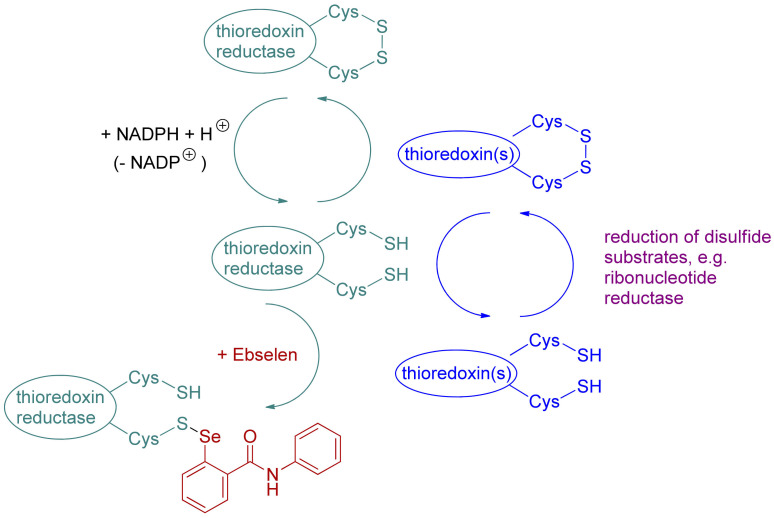
General mechanism of the function of the thioredoxin reductase/thioredoxin system and its inactivation with ebselen.

The mechanism of antibiotic action and the molecular target of ebselen were broadly illustrated by the work of Holmgren’s group. Studies have shown that this compound potently inhibits *Escherichia coli* thioredoxin reductase (*K*_i_ = 0.52 µM) with a competitive and reversible mode of action [21]. Using NADPH, the flavoprotein thioredoxin reductase recycles thioredoxin, a disulfide oxidoreductase, within the system, which catalyzes the reduction of oxidized cysteine and the cleavage of disulfide bonds in multiple substrates (Scheme 1) and thus plays a fundamental role in processes, such as defense against oxidative stress and DNA synthesis [22]. As thiol redox homeostasis is critical for cell viability and proliferation, many pathogenic bacteria lacking support with the glutathione reductase/glutaredoxin system (in general Gram-positive bacteria, for example, *S. aureus* and *M. tuberculosis*, but with certain exceptions, for example, *H. pylori*) are sensitive to the inhibition of thioredoxin reductase [23]. Inhibition causes the accumulation of oxidized thioredoxins, alters the reductions of cellular disulfides, increases oxidative stress, and ultimately leads to cell death. Accordingly, ebselen exhibited a significant effect on drug-resistant strains of *H. pylori* and *M. tuberculosis*, with MIC/MBC (minimum bactericidal concentrations) in the range of 3.13–20 µg/mL [21]. Taking into account the molecular mode of binding, it was hypothesized that the organoselenium compound formed a covalent S–Se bond by opening the ring with thiolate of one of the cysteines of the Cys-Pro-Gly-Cys active site motif (Cys-Xaa-Xaa-Cys, in general, Scheme 1). The transient complex was trapped by reaction with ^14^C-radiolabeled ebselen, indicating Cys135 as the selenation position.

Importantly, the mechanism is highly specific for bacteria. Ebselen is a substrate, not an inhibitor, of mammalian thioredoxin reductase due to its structural and functional dissimilarities from the prokaryotic enzyme [24].

The effect of targeting thioredoxin reductase in Gram-positive and Gram-negative bacteria involving ebselen has also been studied in synergy with other agents [25]. Consequently, antibacterial properties against multidrug-resistant *S. aureus* LT-1 skin infection were improved by a combination of ebselen and curcumin. Somewhat similarly to ebselen, curcumin is a multifaceted molecule that interacts with multiple molecular targets and thus possesses broad antioxidant, anti-inflammatory, antimicrobial, and other activities. First, the minimal inhibition concentration for the organoselenium compound alone was found to be 2.2 mg/mL (8 µM), and treatment with 10-fold MIC triggered the rupture of the cell membrane and wall, flow out of the cytoplasmic fluid, and ultimately the cell death of the pathogen. In a rat model, when treated topically (25 mg/kg), ebselen significantly reduced the mean bacterial count and the expression of host proinflammatory cytokines (tumor necrosis factor-α, interleukin-6, and interleukin-1β). Thus, pathogen eradication helped with the improved healing of damaged skin. The synergistic effect of the inhibition of bacterial growth was achieved for 5 µM ebselen and 10 µM curcumin (0.89 in the Bliss model after 2 h of incubation). Indeed, the combination of both compounds had a significantly stronger inhibitory effect on *S. aureus* thioredoxin reductase [25].

The inhibitory activity of ebselen was also tested with *Bacillus anthracis* thioredoxin reductase (IC_50_ = 1.0 µM) [26]. The potency was comparable to that measured before for the enzyme of *E. coli*. The antibacterial properties of ebselen and its derivatives were evaluated in *Bacillus subtilis*, *Bacillus cereus*, *S. aureus,* and *Mycobacterium tuberculosis*. The minimum inhibitory concentrations found for the parent compound were 0.14 μg/mL, 0.9 μg/mL, 1.1 μg/mL, and 10 μg/mL, respectively, while the minimum bactericidal concentration was up to 1.5 times the MIC. This indicated a general class-dependent mode of action. Although some compounds among the tested derivatives exhibited higher enzyme inhibitory activity (ethylenebis(benzisoselenazolone), for example, IC_50_ = 0.07 µM) than ebselen, they were of comparable potency in cell lines. The toxicity measured against 293T cells from the human embryonic kidney revealed a favorable selectivity ratio (IC_50_/MIC) for ebselen, 240 for *B. subtilis*, 36 for *B. cereus,* and 30 for *S. aureus* [26].

The treatment of *Deinococcus radiodurans*, an extremophilic Gram-positive bacterium, with inhibitors of thioredoxin reductase, including epigallocatechin gallate, auranofin, and ebselen, caused its sensitivity to oxidative stress [27]. Ebselen (10 μM) completely suppressed reductase activity. The alteration of the redox system was associated with cell death of *D. radiodurans* mediated by hydrogen peroxide-induced oxidative stress. The intracellular redox status was further illustrated by metabolomic and proteomic profiling in response to ebselen and radiation treatment [28,29]. The results revealed a change in the composition and concentration of metabolites [28] and a decrease in the expression of essential cellular proteins (e.g., those involved in glycolysis, proteases, peptidases, and peptide transporters) that are necessary for the survival of the bacterium with exposure to ionizing radiation [29].

## 4. Synergy of Ebselen with Silver

The use of silver ions (silver nitrate) and ebselen led to a novel synergistic strategy that selectively targets the bacterial thiol-dependent redox system(s) [30]. The bactericidal effect of ebselen alone was strong in multidrug-resistant Gram-positive bacteria lacking glutathione and glutathione reductase (*vide supra*). However, Gram-negative bacteria, which in most cases possess the backed-up redox system, also appeared to be sensitive to the drug combination. Ebselen decreased the antibacterial concentration of silver against *E. coli* by one order of magnitude. The minimal inhibition concentration of Ag^+^ alone was 42 µM, while the coadministration of 2 µM ebselen reduced the concentration to 4.2 µM [30]. Significantly, no synergistic toxicity was evidenced by the use of 5 µM Ag^+^ and 2.5 µM ebselen in human HeLa cells. The potential of the compound for the formation of *E. coli* colonies and cell viability was further confirmed. The minimal inhibition concentration of Ag^+^ for a variety of multidrug-resistant glutathione-containing Gram-negative pathogens (*K. pneumonia*, *A. baumannii*, *P. aeruginosa*, *E. cloacae* and *E. coli* species) isolated in the clinic was reduced by 4–8 times when ebselen concentrations of 0 µM and 4 µM were compared (without significant effects separately). The drug combination irreversibly inhibited thioredoxin reductase and decreased or depleted protein S-glutathionylation, indicating the disruption of both redox pathways, and caused the elevation of reactive oxygen species [30].

The therapeutic potential of Ag/ebselen treatment against clinically isolated multidrug-resistant strains, uropathogenic *E. coli*, and *A. baumannii*, was evaluated in vitro and in mouse models [31,32]. The results of the studies were consistent and showed the inhibition of thioredoxin reductase activity, reduction of bacterial loads, downregulation of proinflammatory cytokines, and upregulation of reactive oxygen species. In the case of *A. baumannii*, for example, 4 µM ebselen decreased the minimal inhibition concentration of Ag^+^ from 16 µM (alone) to 0.5 µM (in combination) [32]. Ag^+^/ebselen (2 µM/4 µM) showed a synergistic bactericidal effect, while a concentration of up to 80 µM of ebselen was found to be insignificant. The bacterial load in a mouse model of urinary tract infection was significantly reduced. The ELISA showed lower levels of tumor necrosis factor-α, interleukin-6, and interferon-γ [32].

Similar effects, namely, the reduction of glutathione concentration and inhibition of thioredoxin reductase, induced intracellular oxidative stress after synergistic treatment with Ag^+^/ebselen of Gram-negative *Yersinia pseudotuberculosis* [33]. Bacterial loads were significantly reduced, and a pro-immune modulatory effect with low toxicity was observed in vivo.

By condensation of the 4,4′-azodianiline and 2,4,6-triformylphloroglucinol, an advanced covalent organic framework was synthesized to carry thiol-targeting inhibitors [34]. The framework loaded with silver ions and ebselen was designed to be dissociated by azoreductase at the infection sites and to release the active components. The nanodelivery system showed synergistic bactericidal activity with Gram-positive *S. aureus* (MIC = 12.5 μg/mL) and Gram-negative *E. coli* (MIC = 25 μg/mL), which were applied as bacterial models in vitro. When PEG-ylated with methoxypolyethylene glycol amine, the nanoinhibitor demonstrated satisfactory biocompatibility and anti-inflammatory and wound-healing properties in an in vivo infection model in mice with low toxicity to normal cells [34].

Similar to silver ions, silver nanoparticles are strong inhibitors of bacterial thioredoxin reductase and have a synergistic effect on *E. coli* and *S. aureus* in vitro in combination with ebselen [35]. The activity of *E. coli* thioredoxin reductase decreased in a concentration-dependent manner; an 80% inhibitory effect of nanoparticles was observed at a concentration of 0.54 µg/mL. Neither 5 µg/mL silver nanoparticles nor 20 µM ebselen alone showed an inhibitory effect on the growth curve of *E. coli*, while the combination of 0.625 µg/mL and 20 µM µg/mL was completely bactericidal with 9 h of treatment (0.625 µg/mL and 10 µM µg/mL for *S. aureus*) [35].

## 5. Specific Bacterial Protein Targets for Ebselen

Ebselen is reactive to low-molecular-weight thiols, including cysteines in short peptides, such as glutathione [36]. The organoselenium compound is also capable of targeting multiple (catalytic) proteins other than thioredoxin reductase, acting competitively or allosterically. To mention one of the earlier works, ebselen was suggested to inactivate the plasma membrane H+-ATPase [37]. Indeed, this was evidenced as time- and concentration-dependent inhibition for the yeast enzyme. However, the corresponding bacterial enzymes could also serve as reactive thiol donors. Accordingly, the three strains of Gram-positive bacteria (*S. aureus*, *Micrococcus luteus*, and *B. subtilis*) were effectively inhibited (IC_50_ = 2–5 μM); less consequently, no significant effect was observed for three Gram-negative bacteria (*E. coli*, *P. aeruginosa*, and *Salmonella enteritidis*, IC_50_ > 80 μM). This result indicated that interference with microbial targets. either commonly present (e.g., thioredoxin reductase) or specific, was complex and characteristic of the microorganism. Some specific examples are discussed below.

### 5.1. Clostridium Difficile Toxins

Ebselen was identified as a molecule capable of protecting cells from damage caused by toxins produced by pathogenic strains of *Clostridium difficile* [38], a Gram-positive anaerobe that is the leading cause of nosocomial diarrhea [39]. Two homologous glycosylating toxins, A and B, are the main virulence factors of *C. difficile*, which is responsible for the destruction of human colonocytes and the production of fluid secretion and tissue damage, usually after antibiotic treatment [40]. Controlling bacterial pathogens by targeting virulence factors is a relatively rare approach. In this case, Bender et al. demonstrated that ebselen inhibited activity and, consequently, prevented pathologies caused by *C. difficile* pathologies in an in vivo infection model [38]. In detail, ebselen was identified as a very potent inhibitor of the cysteine protease domain of *C. difficile* toxin B (IC_50_ = 6.9 nM) among a set of clinically safe candidates. These compounds were used in cell-rounding assays, which involved their coadministration with the active full-length toxin on human foreskin fibroblasts. Ebselen efficiently blocked cell rounding with EC_50_ = 20.5 nM. A protective effect was still observed when cells were preincubated with ebselen, washed, and subsequently submitted to the action of purified toxin B. These data indicated the intracellularly active pool of ebselen. Finally, toxin-mediated cytopathology was inhibited in clinical samples (EC_50_ = 372 nM), where both toxin A and B were present [38]. Ebselen also induced the loss of function of toxin B in an in vivo model. Mice fully survived a lethal dose when pretreated with 100 nM ebselen, with no symptoms of vitality loss. The treatment moderated histopathological effects in a model of post-antibiotic *C. difficile* infection. Vascular and epithelial damages were not observed, while the scores for cell infiltration, submucosal edema, and mucosal hypertrophy were considerably reduced [38]. In a subsequent study in animal models, orally administered ebselen confirmed a decrease of tissue damage caused by *C. difficile*, and the risk of reoccurrence of the infection [41]. The improved recovery of microbiome diversity from persistent dysbiosis after the antibiotic treatment of infected mice was also demonstrated. Thus, ebselen, alone or in combination with antibiotics, was validated as a therapeutic approach for infected patients, and a preventive treatment for those at high risk of infection.

Disclosing the molecular mechanism of action, Beilhartz et al. provided evidence that the antitoxin protective activity of ebselen was not (only) achieved by inhibiting the autocleavage domain [42]. The activation of this triggers the release of the glucosyltransferase domain of toxin B. Ebselen decreased the ability of the latter to transfer glucose from uridine 5′-diphosphate-glucose to human Rac1, a GTPase, in a dose-dependent manner. The covalent binding of ebselen to Rac1 at three positions (Cys105, Cys157, and Cys178) prevented transfer and indirectly inhibited glucosyltransferase activity and toxin B-induced cell rounding [42].

A more general influence of ebselen on the virulence potential was recently suggested [43]. The study reevaluated the lack of antimicrobial properties against *C. difficile*, compared with typical activity reported for other species. Indeed, the compound showed substantial bactericidal action by inhibiting most of the 12 tested *C. difficile* strains (MIC in the range of 2–8 μg/mL) when measured in brain heart infusion agar. The MBC of ebselen was 16 mg/mL (8×MIC), while vancomycin killed bacteria at 4 to 8 mg/mL (8 to 16×MIC). Transcriptome analysis showed characteristic alterations in redox processes, cysteine metabolism, and the NAD^+^/NADH ratio, which had multiple negative impacts on toxin production and sporulation. Significantly, Ebselen inhibited the growth of some representatives of gut flora, for example *Actinomyces viscosus*, *Lactobacillus crispatus*, *Lactobacillus johnsonii*, *Fusobacterium nucleatum*, and *Fusobacterium periodonticum* (MIC = 2–32 mg/mL) [43].

### 5.2. Urease

Among an extensive series of 1,2-benzisoselenazol-3(*2H*)-ones and their open-cycle diselenide derivatives, ebselen was found to inactivate the most efficient *Sporosarcina pasteurii* and *H. pylori* urease [44], a dinickel metallohydrolase [45,46]. *K*_i_ values were in the nanomolar range of *K*_i_ = 2.11 nM and 226 nM. The inhibitor showed a covalent and irreversible mode of binding; thus, the proposed mechanism of action involved modification of a cysteine residue. Cys322 (for *S. pasteurii*), which is conserved among bacterial enzymes and located at the entrance of the active site and involved in conformational changes upon substrate binding, is the most evident candidate. As illustrated by molecular modeling, the formation of the S–Se covalent adduct was assisted by favorable interactions and, in particular, a hydrogen bond between the NH group of the ligand and C=O of the modified Cys322 and π−π stacking of the *N*-phenyl ring of ebselen and the imidazole of the neighboring His323. The organoselenium compound confirmed its ability to penetrate the cell membrane and its antiureolytic activity in whole-cell *E. coli* and *H. pylori* models in vitro [44].

### 5.3. M. tuberculosis Antigen 85 and l,d-Transpeptidase 2

**Scheme 2:**
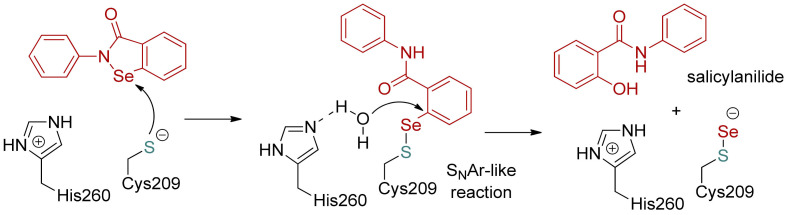
The proposed mechanism of selenation of a cysteine residue and aging of the preliminary adduct, including the opening of the ebselen isoselenazolone ring and the hydrolytic release of salicylamide, adapted to antigen 85C based on the structure of SARS-CoV-2 M^pro^ [49].

The antigen 85 complex and l,d-transpeptidases have been identified as attractive targets for the development of new antimicrobials against *M. tuberculosis*, as these proteins play important specific roles in the resistance and virulence of the bacterium. Ebselen was shown to be a potent inactivator of both enzymes; furthermore, the mechanistic aspects of inhibition were provided by resolving the crystal structures of ligand-protein complexes. The antigen 85 complex (three homologous mycolyltransferases) catalyzes the transesterification of mycolic acids (long, α-branched, β-hydroxy fatty acids) to form mycolylarabinogalactan and trehalose dimycolate, which are components of leaflets of the mycobacterial outer membrane [47]. Ebselen inhibited antigen 85C with an observed *K*_i_ of 63 nM [48]. As evidenced by the crystal structure, upon binding, ebselen altered the structure of the transferase active site by disrupting the hydrogen bond network formed by the catalytic triad (S124, E228, and H260). Surprisingly, the density that represents the ebselen expected in the loop, which connects C209 (the obvious target for ebselen) and helix α9, is lacking. The interpretation of this observation provided by the authors included a structural disorder and/or rupture of the S–Se bond by ionizing radiation [48]. Recently, an interesting alternative for the inactivation of M^pro^, the main protease of SARS-CoV-2, with ebselen was presented [49]. The spatial proximity of histidine and cysteine, later modified by reaction with ebselen, induced acid-base-catalyzed aging of the initial complex. This process involved S_N_Ar-like nucleophilic hydrolysis of the adduct and the release of salicylanilide and the selenated enzyme [49]. In antigen 85C, His260 appears to be conveniently located against Cys209 (7.25 Å between the α carbon atoms) to perform corresponding catalysis [50], while the density representing selenium in the tentative product could be confusing for interpretation in the disordered region. We believe that such a mechanism (Scheme 2) is of general significance and should be considered for the reactions of ebselen with protein targets.

**Figure 2:**
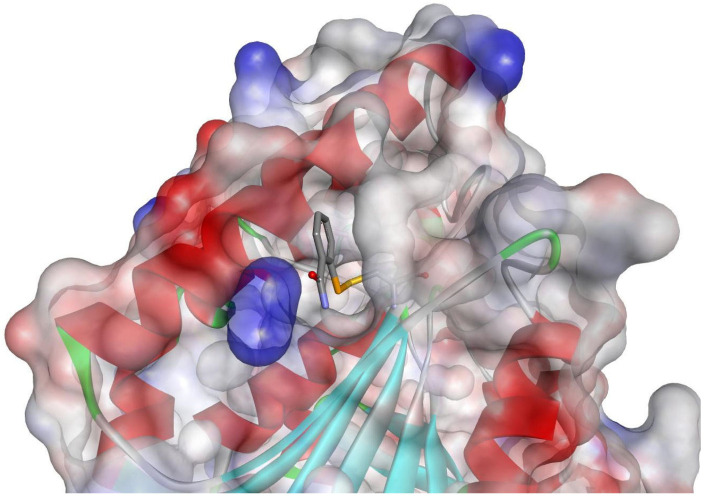
Binding mode of ebselen with antigen 85C (Protein Data Bank (PDB): 4QDU). The inhibitor reacted with C209 is shown as sticks, while the enzyme is shown as a ribbon. The surface of the protein is colored according to the charge.

**Figure 3:**
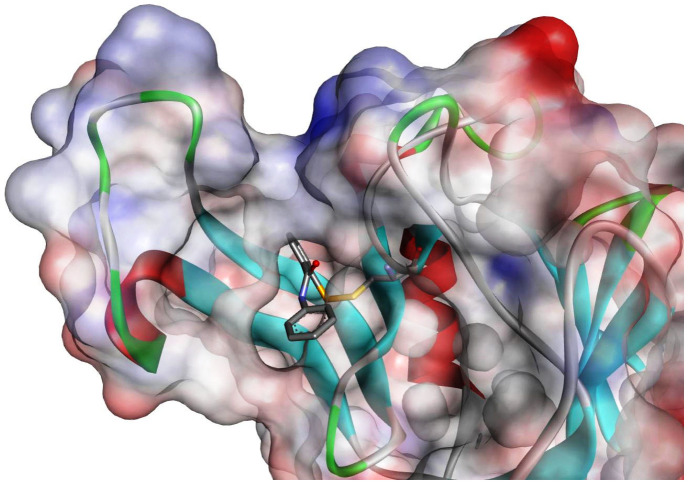
Binding mode of ebselen with l,d-transpeptidase 2 of *M. tuberculosis* (PDB: 6RRM). The inhibitor reacted with Cys354 in two conformations.

Nevertheless, the following studies were performed at a wavelength that decreased the absorbance of the selenium atom to avoid oxidation and revealed the typical covalent modification of C209 [51]. Although the structure of ebselen was partially disordered, the open form of the complete molecule was evident (Figure 2). Subsequent structures of two complexes with benzisoselenazolone derivatives further confirmed this mode of binding and its critical influence on conformational changes and the stability of antigen 85C [52].

l,d-transpeptidases form cross-links in the cell wall between two residues of *meso*-diaminopimelic acids [53]. Ebselen inhibited l,d-transpeptidase 2 with IC_50_ = 0.36 μM (without preincubation) and IC_50_ = 0.143 μM (after 60 min of preincubation) [54]. When soaked with the inhibitor, the enzyme was found to form a typical covalent adduct with ebselen via Cys354 thiolate (Figure 3). Two different conformations were refined in chain A, while a single conformation was refined in chain B. Both conformations are characterized by extensive hydrophobic interactions with the residues that form the movable active-site loop (Val333, Phe334, and His352) and the hydrophobic pocket around Cys354 (Met303, Tyr308, Tyr318, and Thr320). The proximal phenyl ring of the ligand is mainly involved in π-stacking with Tyr318, while the location of the distal ring (*N*-Ph) depends on the conformation [54].

### 5.4. New Delhi Metallo-β-Lactamase-1

New Delhi metallo-β-lactamase-1 (NDM-1) is classified in subclass B1 of the metallo-β-lactamases based on the amino acid sequence and the mode of coordination of zinc atoms at its active site. As NDM-1 is capable of accommodating and inactivating the majority of β-lactam antibiotics and no inhibitors have been introduced in the clinic, it has become a notorious bacterial resistance factor [55]. The flexible active site binds to the carboxylate of β-lactam using two zinc ions that are coordinated by surrounding amino acid residues, mainly histidines, and one cysteine (Cys221 in NDM-1 of *E. coli*) [56]. The last residue of the enzyme was suggested to be targeted with ebselen. The study involved the use of an *E. coli* strain that carried a plasmid that encoded full-length NDM-1. Combining ebselen with ampicillin or meropenem (in a ratio of 1.3–1.4 to 1) reduced the minimum inhibition concentration 16 and 128 times, respectively, compared to the only antibiotic action [57]. An additional increase in the concentration of ebselen allowed the achievement of submicromolar values for meropenem (0.65 µM). The effect of sensitization of the NDM-1-positive strain of *E. coli* to meropenem was attributed to the irreversible or slowly reversible binding of benzisoselenazolone to the enzyme. Inactivation kinetic parameters were determined to be *K*_i_ = 0.38 µM and *k*_inact_ = 0.034 min^−1^ (*k*_inact_/*K*_i_ = 1.5 mM^−1^s^−1^). The ESI-MS analysis of native NDM-1 incubated with ebselen resulted in a clear peak that included the sum of mass of the NDM-1 protein, ebselen, and a zinc ion [57]. The release of the second zinc ion supported the involvement of Cys221 in the formation of the S–Se complex, which disrupted the metal coordination system. The inhibitory effect of ebselen could be attenuated with thiols, such as DTT, in competition assays.

Further studies of the structure-activity relationship between 1,2-benzisoselenazol-3(2*H*)-ones and carbapenem-resistant Enterobacteriaceae producing New Delhi metallo-β-lactamase led to the identification of another promising compound among 46 candidates [58]. The 2-(*tert*-butoxycarbonyl)ethyl-derived inhibitor inactivated NDM-1 following *pseudo*-first-order kinetics with parameters of *K*_i_ = 17 µM and *k*_inact_ = 0.068 min^−1^ (*k*_inact_/*K*_i_ = 0.067 mM^−1^s^−1^). Importantly, when combined with this compound, meropenem regained antimicrobial potency against an NDM-1-positive strain, showing a significant synergistic effect in a *Galleria mellonella* larvae infection model and low cytotoxicity with eukaryotic cells.

**Scheme 3:**
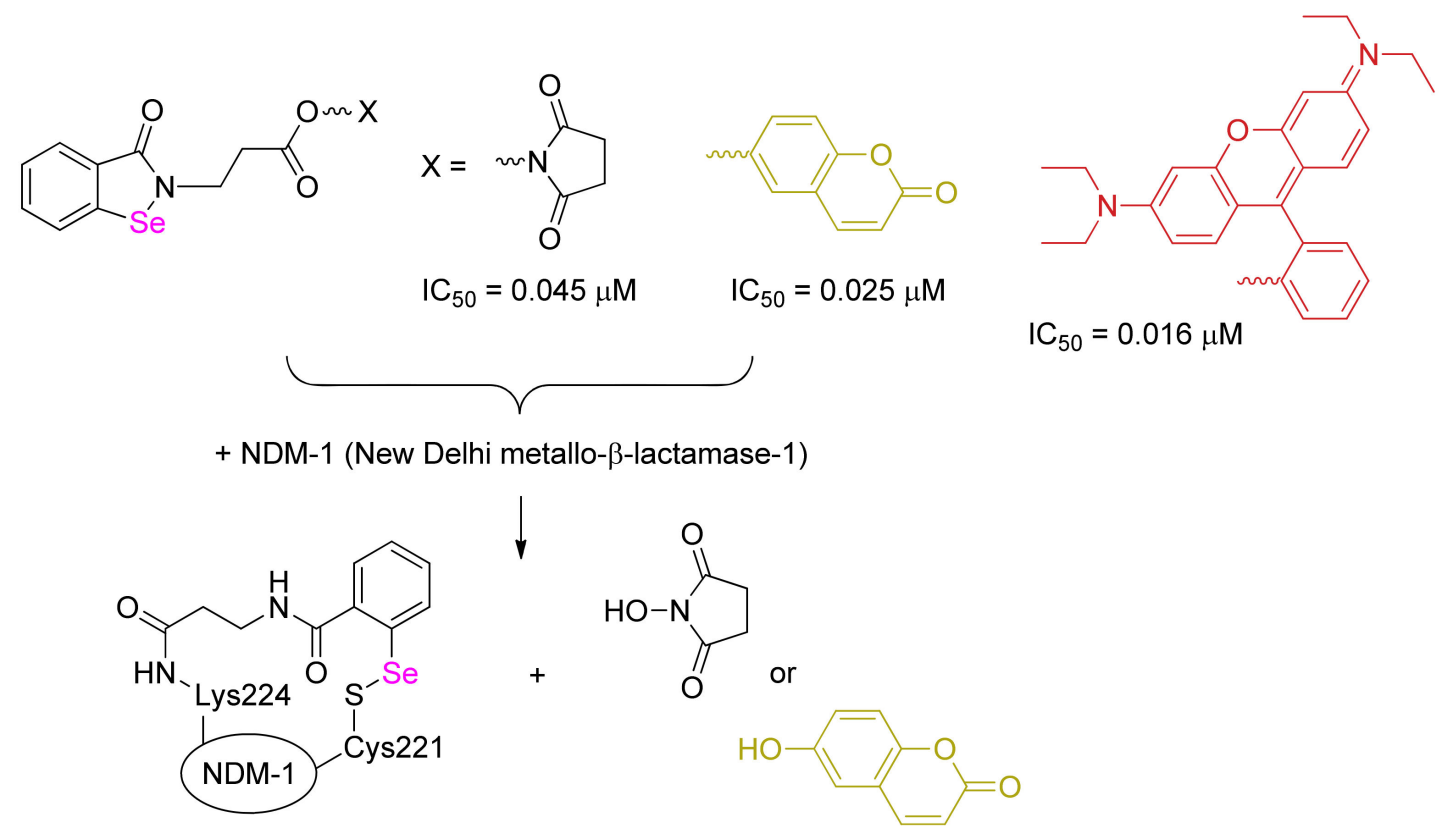
Potent and specific dually acting covalent inhibitors and a fluorescent probe of New Delhi metallo-β-lactamase-1; the structures are based on 2-(2-carboxyethyl)-1,2-benzisoselenazol-3(2*H*)-one.

This structural scaffold was also used to construct advanced derivatives, potent and specific covalent inhibitors, or fluorescent probes of NDM-1 and related lactamases. Succinimide and coumarin-based esters were supposed to act by dually modifying Cys221 by selenation and Lys224 via acylation, with the latter associated with the release of the florescent portion (Scheme 3) [59]. The use of rhodamine B led to the compound that labeled the single site (Cys221).

Activity similar to that shown by ebselen with NDM-1 was also evidenced for its sulfur counterpart, ebsulfur [60,61]. *N*-Phenylbenzisothiazol-3(2*H*)-one exhibited the highest affinity among the 18 tested analogs and inhibited lactamase by the covalent mechanism with IC_50_ = 0.16 μM. It also restored the antibacterial activity of cefazolin against *E. coli*-producing NDM-1 by reducing the MIC from 256 μg/mL to 1 μg/mL at a concentration of 16 μg/mL. Similar to ebselen, the benzisothiazolone scaffold conjugated with rhodamine B through the carboxylic acid linker was used to construct a fluorescent probe for effective labeling and imaging lactamase in vitro and in vivo [60].

The final examples mentioned above [58,59,60,61] encompass applications not precisely of the ebselen fundamental structure but its modifications. In fact, the molecular architecture of ebselen allows multidirectional functionalization and diversification [6]. These modified compounds share a common mechanism of action, but their reactivity and selectivity may be suitably tuned. Apparently, they have also been thoroughly studied for their antimicrobial properties. Although it is beyond the scope of this account, we wish to mention the most representative arbitrarily selected contributions in this field: diversification and functionalization of the phenyl rings of the lead compound [62,63,64], sulfur analogs (replacing the selenium atom) [60,65], and heteroatom-oxidized derivatives [66]. These cases illustrate the fact that ebselen remains an inspiring lead compound in the structural exploration of new antimicrobial treatments.

## 6. Conclusions

In 2017, *E. coli*, *S. aureus*, *K. pneumoniae*, *S. pneumoniae*, *A. baumannii*, and *P. aeruginosa* (*ESKAPE*) were designated by the WHO as the six main bacterial pathogens responsible for fatal cases associated with increased antibiotic resistance [67]. In recent years, other multidrug-resistant infections have emerged rapidly, e.g., tuberculosis, the single disease that causes at least 1 million deaths per year, with a growing number caused by multidrug-resistant *M. tuberculosis* [68]. Consequently, understanding the mechanisms of bacterial resistance to commonly used antimicrobials is of particular importance, while the implementation of new approaches for the treatment of persistent pathogens is another priority. As the development of innovative antimicrobials has a long-lasting gap, alternative strategies, such as targeting biofilm formation or bacteriophage therapy, are proposed as alternatives. The identification of new activities for existing pharmaceuticals (repurposing/repositioning of drugs) also seems to be an attractive option that follows the urgency and economical aspects of efficient treatments. Although not yet approved by the FDA, ebselen shows several advantageous features in this context. Despite reactivity with multiple targets, it is considered safe as its cytotoxicity with human cell lines is at a reasonable level; consequently, it reached phase II clinical trials for the treatment of stroke and prevention of hearing loss [69,70,71].

In general, ebselen acts as a covalent inhibitor that reacts with various protein thiols to form S–Se-bonded irreversible complexes. Thioredoxin reductase, specifically bacterial not mammalian, is the most recognized molecular target for this compound. The inhibition of this enzyme blocks the system of disulfide reduction in multiple substrates that alters the biosynthesis of DNA and essential cellular proteins, influences the redox status of the cell, and induces oxidative stress (Table 1). The bacterial species that do not involve the alternative glutathione reductase system, which are mostly Gram-positive, including clinical isolates of multidrug-resistant *ESKAPE*, are mainly sensitive to ebselen treatment. Nevertheless, other pathogens can also be targeted by suitable combinations of ebselen with an existing drug, the action of which typically shows synergism.

In addition to the suppression of cellular protein synthesis by the inhibition of thioredoxin reductase, ebselen directly inactivates multiple bacterial enzymes, toxins, and resistance factors. The decreased activity of these factors reduces virulence and sensitizes pathogens to classical antimicrobials.

Ebselen has advantageous properties in the eradication and inhibition of the formation of bacterial biofilms. It was also found that it does not generate bacterial resistance to its application. Together, these features suggest ebselen should be considered in a prospective antimicrobial treatment but for the coadministration for particular specific infections rather than as a broad-band antibiotic. For example, the synergistic action of ebselen with topical antimicrobials seems to be promising for the prophylaxis and treatment of *S. aureus*-infected wounds and skin infections acquired through public health care [13,15]. Sensitization to common antibiotic agents by inhibiting New Delhi metallo-β-lactamase-1 is also a promising alternative to eliminate species that produce this resistance factor [55,56]. Finally, due to substantial antiureolytic and antitoxin activity, ebselen shows the potential to be applied to infections caused by *H. pylori* and *M. tuberculosis* [44,48].

## Figures and Tables

**Table 1:** Summative effect of ebselen on pathogenic bacteria and associated infections.

Activity of Ebselen	
Intracellular mechanisms	Inactivation of bacterial thioredoxin reductase Accumulation of oxidized thioredoxins Upregulation of reactive oxygen species Disruption of cellular redox pathways (downregulation of DNA synthesis and expression of cellular proteins) Inhibition of bacterial proteins, toxins, and virulence factors
Extracellular mechanisms	Inhibition of biofilm formation (degradation of the polymeric layer and attenuation of quorum sensing) Downregulation of expression of host proinflammatory cytokines
Therapeutic effects	Bactericidal properties (alone and in synergic combination with known antimicrobials) Sensitization to common antimicrobials Recovery of microbiome diversity after antibiotic treatment Host immune response modulation

## Data Availability

Not applicable.
